# Cooling aerosols and changes in albedo counteract warming from CO_2_ and black carbon from forest bioenergy in Norway

**DOI:** 10.1038/s41598-018-21559-8

**Published:** 2018-02-19

**Authors:** Anders Arvesen, Francesco Cherubini, Gonzalo del Alamo Serrano, Rasmus Astrup, Michael Becidan, Helmer Belbo, Franziska Goile, Tuva Grytli, Geoffrey Guest, Carine Lausselet, Per Kristian Rørstad, Line Rydså, Morten Seljeskog, Øyvind Skreiberg, Sajith Vezhapparambu, Anders Hammer Strømman

**Affiliations:** 1 Industrial Ecology Programme and Department of Energy and Process Engineering, Norwegian University of Science and Technology (NTNU), Norwegian, Norway; 2 SINTEF Energy Research, Norwegian, Norway; 3 Norwegian Institute of Bioeconomy Research, Norwegian, Norway; 4 National Research Council Canada, Ontario, Canada; 5 Norwegian University of Life Sciences, Norwegian, Norway

## Abstract

Climate impacts of forest bioenergy result from a multitude of warming and cooling effects and vary by location and technology. While past bioenergy studies have analysed a limited number of climate-altering pollutants and activities, no studies have jointly addressed supply chain greenhouse gas emissions, biogenic CO_2_ fluxes, aerosols and albedo changes at high spatial and process detail. Here, we present a national-level climate impact analysis of stationary bioenergy systems in Norway based on wood-burning stoves and wood biomass-based district heating. We find that cooling aerosols and albedo offset 60–70% of total warming, leaving a net warming of 340 or 69 kg CO_2_e MWh^−1^ for stoves or district heating, respectively. Large variations are observed over locations for albedo, and over technology alternatives for aerosols. By demonstrating both notable magnitudes and complexities of different climate warming and cooling effects of forest bioenergy in Norway, our study emphasizes the need to consider multiple forcing agents in climate impact analysis of forest bioenergy.

## Introduction

Bioenergy from forest biomass is renewable and a potential climate change mitigation solution. Least-cost and sustainable pathways to climate stabilization as envisaged by energy scenario literature typically involve significant increases in the use of forest bioenergy in decades to come, often in conjunction with use of carbon capture and storage in order to achieve negative carbon dioxide (CO_2_) emissions^1–3^. Unlike other renewable energy sources, bioenergy is a combustible fuel energy source, exhibiting characteristics (e.g., possibility for energy storage and transport by road, rail or water) that can help to yield diversified low-carbon energy systems.

Assessing the climate change impacts of forest bioenergy is complex, because it involves a multitude of climate forcing agents. Traditionally, and still the case in prevailing climate policy frameworks and many assessment models^4^, bioenergy is designated as carbon neutral. As is recognized in literature^5–7^ and recent legislative efforts^8^, this paradigm does not consider that there can be an initial period where carbon accumulates in the atmosphere because emissions from biomass combustion occur at a faster rate than CO_2_ uptake by vegetation re-growth. This temporal asymmetry induces a perturbation of the global carbon cycle and climate system, which result in a temporary warming effect^9^. Further, burning biomass leads to emissions of near-term climate forcers (NTCFs) that exert warming or cooling effects over a relatively short time period. Notable NTCFs are black carbon (BC), organic carbon (OC), carbon monoxide (CO), volatile organic compounds (VOC), mono-nitrogen oxides (NO_x_) and sulphur oxides (SO_x_)^10^. The net climate impacts of NTCFs are the result of many complex effects with different temporal evolutions at play, and their quantification is subject to large uncertainties^11^. Some of these pollutants (NO_x_, CO, VOCs) are precursors to the formation of tropospheric ozone, a powerful GHG. Others are primary aerosols (BC, OC) or precursors to secondary aerosols (NO_x_, SO_x_) that influence climate in various ways. For example, OC and SO_x_ result in an increase in scattering of solar radiation, and hence cool the climate system. Finally, wood harvest-induced alterations of surface albedo and other biophysical factors affect climate^12,13^. The magnitude and direction (warming or cooling) of biophysical climate effects vary and are strongly site-specific^14,15^. Alteration of surface albedo directly affects the global radiation balance^12,16^, and is particularly important in areas with seasonal snow cover^17,18^.

Life cycle assessment (LCA) is a widely applied tool for assessing the climate impacts of energy options^19,20^. The strength of LCA is that it takes into consideration not only direct emissions from combustion, but also emissions occurring upstream in supply chains. Climate impact assessments in existing bioenergy LCAs mainly focus on emissions of GHGs (e.g., refs^21,22^). However, there is a growing literature showing that surface albedo changes^23–27^ and NTCFs^28,29^ can be important in shaping the climate impacts of bioenergy, and hence should be included in climate impact assessment^30^. To our knowledge, no bioenergy LCA studies undertaken at a national scale with technology-level detail have simultaneously examined the climate effects of supply chain GHGs, biogenic carbon fluxes, NTCFs and surface albedo changes. These effects can be consistently included in LCA by employing emission metrics that translate the effects to common units, such as CO_2_-equivalents^31^.

Here, we assess the balance of climate cooling and warming effects of wood stove and district heating forest bioenergy in Norway. The 100-year global warming potential (GWP) is used to characterize and aggregate climate effects of different climate forcers. We integrate a mapping of regional wood harvest and bioenergy supply chains in Norway, sets of emission factors established from own experimental data and other sources, own computed geographically explicit GWPs for biogenic carbon fluxes and changes in surface albedo, and GWPs for GHGs and NTCFs from the IPCC (see Methods). In this way, we are able to present a first-of-its-kind elucidation of the magnitudes of and balance between climate warming and cooling effects from multiple forcing agents associated with forest bioenergy at a national level. We find that cooling from OC and albedo changes offset 60–70% of warming from BC and GHGs, leaving net total warming effects of 69 kg CO_2_e MWh^−1^ for district heating and 340 CO_2_e MWh^−1^ for wood stove heating on average. These magnitudes are significantly lower than (for district heating) or comparable to (for wood stoves) GHG emissions associated with fossil oil-based heating. They appear comparable or large in comparison to life cycle GHG emissions of heating based on renewable electricity. Estimated total cooling and total warming from forest bioenergy are both substantial: total cooling effects are in the range of 200–400 kg CO_2_e MWh^−1^ and warming effects 300–800 kg CO_2_e MWh^−1^.

## Results

### Forest biomass-to-bioenergy systems

Norway is a country with vast forest resources. We focus on two important applications of forest bioenergy in Norway: wood-burning stoves and wood biomass-based district heating. Wood stove bioenergy represents a major application of forest biomass and source of BC and OC emissions in Norway^32^. In comparison to stoves, forest biomass-fired plants for district heating are a larger-scale application with more pollution abatement.

We model four kinds of residential firewood stoves, classified as either old or new, clean-burning stoves, and as operating at either partial load or nominal load. We distinguish five categories of combustion plant sizes. The various stove and combustion plant types are distinguished by different efficiency and emission factor values, considering a Norway mix of biomass fuel types (see Methods). Table 1 gives estimated wood harvest amounts attributed to the technologies under study, and heat delivery and thermal efficiency values for year 2010. Total wood stove bioenergy production is roughly 80% greater than district heating bioenergy. Stove bioenergy is largely produced from partial load operation (69%, with the remainder coming from nominal load). Nearly half of the district heating bioenergy is produced by plants in the 1 < 10 MW capacity range. Attributed harvest amounts exceed the amounts of final fuel because of losses and burning along the supply chain to produce biofuels. At the same time, waste wood, overall constituting 9% of the feedstock for district heating, is assumed to not require any harvest. A caveat of our analysis is that harvest quantities are attributed to firewood under certain simplifying assumptions necessary due to gaps in available data. In particular, we model private harvest and net import as if it occurs in Norway with a county distribution equal to the distribution of firewood consumption (see Methods for details).

**Table 1:** Annual attributed wood harvest, annual heat output and combustion plant thermal efficiency values for district heating and residential wood stoves in Norway (year 2010). DH = district heating; MW = megawatt. Combustion plant efficiencies vary depending on fuel type.

	Attributed harvest (1000 dry t year^−1^)	Heat output (GWh year^−1^)	Thermal efficiency (%)
Bio DH < 1 MW	60	235	82–83
Bio DH 1 < 10 MW	270	1100	85–86
Bio DH 10 < 20 MW	120	500	89–91
Bio DH 20 < 50 MW	36	157	91–93
Bio DH > 50 MW	65	291	93–95
Stoves, old, partial load	340	1180	67
Stoves, old, nominal load	180	610	65
Stoves, new, partial load	430	1610	72
Stoves, new, nominal load	180	659	69

**Figure 1:**
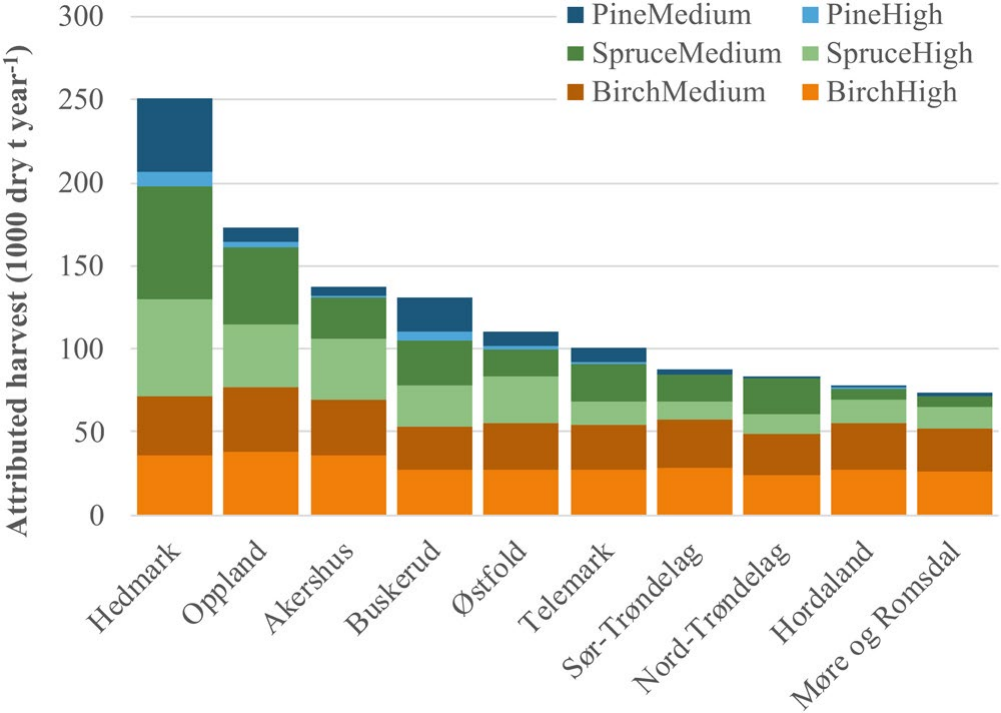
County breakdown of total annual wood harvest attributed to district heating and wood stove bioenergy in Norway (year 2010). Top ten counties in terms of total attributed harvest are shown. Stacked categories represent species (birch, spruce, pine) and forest productivity class (high, medium). Hedmark, Oppland, Akershus, Buskerud, Østfold and Telemark are in south-east Norway; Sør-Trøndelag and Nord-Trøndelag are in central Norway (north of south-east part); and Hordaland and Møre og Romsdal are in coastal west Norway.

Breakdowns per wood classes of forest harvest attributed to bioenergy are shown in Fig. 1, distinguishing the ten most important counties in terms of harvested amount (covering 73% of Norway total), tree species and forest productivity classes. The top six counties (53% of Norway total) are all in the south-eastern part of Norway, a region with intensive forest management and characterized by much more snow cover than the coastal western part. It is also the region with the largest forest resource potentials^33^. Estimated birch harvest is fairly constant across counties, as birch is predominantly (84%) used for firewood and firewood demand is similar across counties. Spruce and pine harvest is comparatively high in the south-east; this is connected to harvest for district heating.

### Global warming potentials (GWPs)

100-year GWP values for well-mixed GHGs (excluding CO_2_ of biogenic origin) and NTCFs are obtained from the fifth IPCC Assessment Report^10^. We use GWP values that include climate-carbon feedbacks for non-CO_2_ gases. This is in line with recent UNEP-SETAC guidelines for LCA^30^ and supported by the conclusion of the IPCC that including climate-carbon feedbacks “likely” (page 714 in ref.^10^) yields better estimates, albeit with higher uncertainty^10^. We employ GWP factors for six NTCFs (black carbon, BC; organic carbon, OC; carbon monoxide, CO; volatile organic compounds, VOC; mono-nitrogen oxides, NO_x_; and sulphur oxides, SO_x_) and estimates of uncertainty intervals (±standard deviation) as given by the UNEP-SETAC^30^ on the basis of ranges presented by the IPCC^10^.

**Figure 2:**
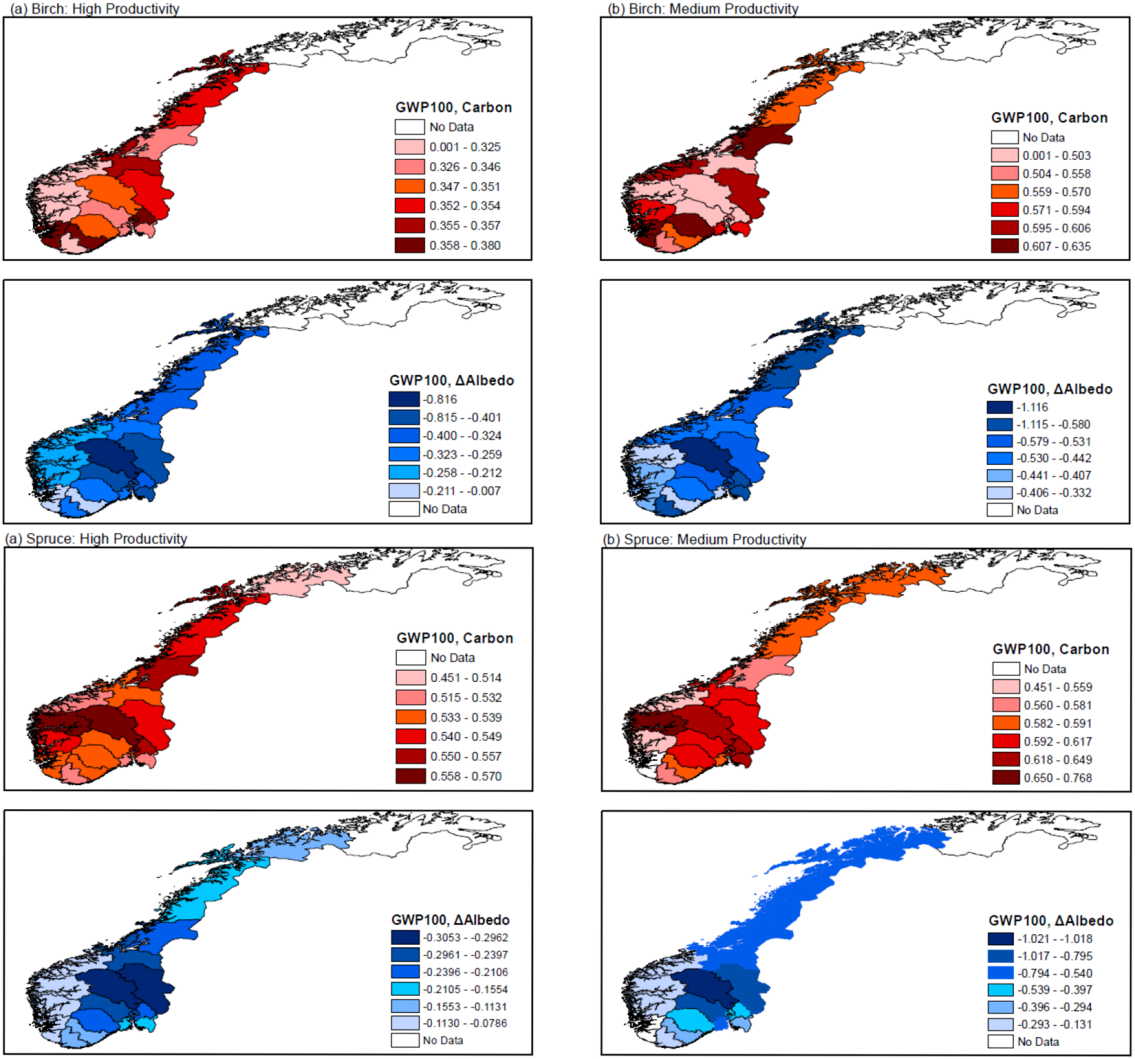
Global warming potentials calculated with a 100-year time horizon (GWP100) for CO_2_ emissions and changes in surface albedo associated with birch and spruce forest bioenergy in Norway. Plots in left and right columns are for high and medium forest productivity classes, respectively. GWP values are expressed in kg CO_2_e per kg of CO_2_ emissions from biomass combustion. The maps were created by using ArcMap 10.3 software (http://desktop.arcgis.com/en/arcmap/).

For biogenic CO_2_ and surface albedo, we compute GWP factors for the nineteen Norwegian counties, three tree species (birch, spruce and pine) and two forest productivity classes (see Methods). Figure 2 shows the GWP values for CO_2_ emissions from biomass combustion and post-harvest albedo dynamics for birch and spruce (results for pine are available in Supplementary Fig. S1).

GWP for CO_2_ emissions from biomass combustion are relatively smaller for forests with relatively higher productivity, as the resource turnover time is shorter^34^. Under the same site class, values tend to be higher in mountainous areas and at increasing distance from the coast. Values for deciduous species (birch) are lower than those for coniferous species (spruce and pine), because birch plantations have significantly shorter turnover times (about 60–70 years against 90–100 years). GWP for changes in surface albedo are lower (that is, lower on a negative scale) for site classes with lower productivity. This is because the longer a forest takes to regrow the more sustained is the post-harvest change in surface albedo over time, because the snow-masking effect of canopy closure is delayed if compared to a high productive site. Albedo GWP factors are particularly significant in the central mountainous areas of the country, mainly owing to abundant snowfalls. Further, they tend to be more significant for deciduous trees than for coniferous species because of the defoliation process of birch-tree types in wintertime, which increases the fraction of snow-covered land exposed to solar radiation.

Net GWP values, meaning the combined contributions from carbon GWP and albedo GWP, vary for the different counties, tree species, and site classes (supplementary Fig. 2). For pine and spruce with high productivity, the carbon contribution dominates and the net GWP values are positive for all counties. For birch, in some counties contributions from albedo dominate even with high productivity, resulting in net negative values of GWP.

### Climate warming and cooling effects

**Figure 3:**
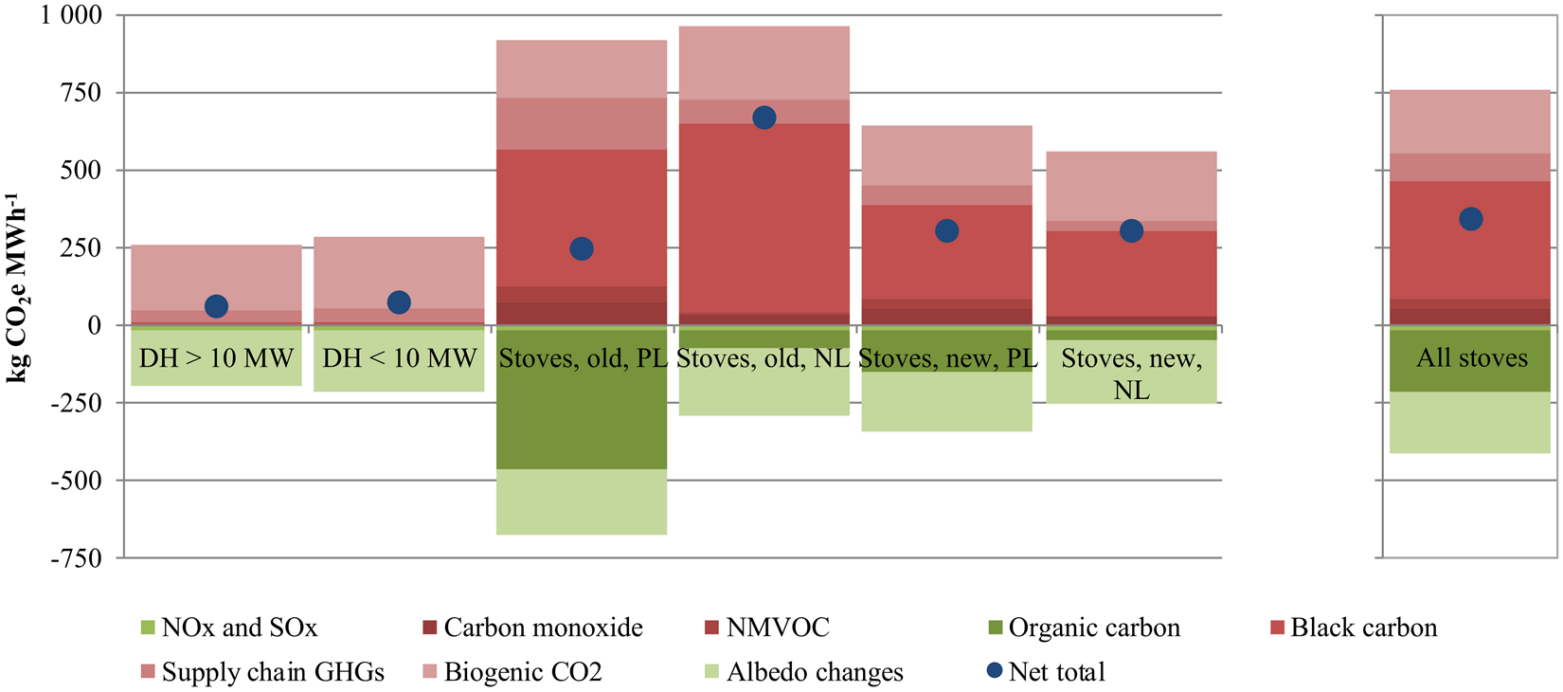
Total annual impacts per unit heat output for wood stove and district heating bioenergy. Left panel shows a total of six wood stove and combustion plant categories separately; right panel shows total across all stove and plant categories. DH = district heating; PL = partial load; NL = nominal load.

Table 2 shows climate impacts per unit of heat output, broken down by six categories of climate forcers. Figure 3 presents a visual display of the results. We find that several climate forcing agents contribute to comparable degrees to the overall climate warming and cooling impacts, with a combined, net effect that is warming. Considering all climate alterations, combined warming amounts to 760 kg CO_2_e MWh^−1^ for stove heating and 270 kg CO_2_e MWh^−1^ for district heating; similarly, combined cooling are 420 kg CO_2_e MWh^−1^ and 210 kg CO_2_e MWh^−1^. Thus, we are left with a net warming of 340 kg CO_2_e MWh^−1^ for stove heating and 69 CO_2_e MWh^−1^ for district heating. By comparison, direct and supply chain GHG emissions associated with delivering electricity to consumers connected to a distribution grid in Norway has been estimated to 42, 190 or 580 kg CO_2_e MWh^−1^, depending on whether Norwegian, Nordic or European electricity is assumed^35^. If assuming the same electricity is used to drive a heat pump with a seasonal coefficient of performance of 3, the emissions would be 14, 65 or 190 kg CO_2_e MWh^−1^. By further comparison, one prospective study estimates a carbon footprint of European electricity production in 2030 of roughly 100 kg CO_2_e MWh^−1 ^^20^ in a 2 °C mitigation scenario. The carbon footprint of burning fossil oil is 320 kg CO_2_e MWh^−1^^ 36^, or 360 kg CO_2_e if considering a 10% distribution loss as assumed in the present study for bioenergy district heating (see Methods).These comparisons are intended to aid interpretation of the magnitude of the estimated bioenergy impacts, but bearing in mind that the carbon footprints for electricity and oil options do not consider biogenic CO_2_, albedo or NTCFs.

**Table 2:** Climate cooling and warming effects by climate forcers and technologies. PL = partial load; NL = nominal load. Numbers are rounded to two significant digits.

	District heating	Stoves, old, PL	Stoves, old, NL	Stoves, new, PL	Stoves, new, NL	Stoves, combined
**Climate cooling per unit of heat output (kg CO**_**2**_ **e MWh** ^**−1**^ **)**						
Albedo change	−190	−210	−220	−200	−200	−200
Organic carbon	−1.7	−450	−57	−130	−34	−200
NO_x_	−15	−12	−12	−11	−12	−12
SO_x_	−1.2	−5.3	−5.4	−5.2	−5.1	−5.2
Total cooling	−210	−680	−290	−340	−250	−420
**Climate warming per unit of heat output (kg CO**_**2**_ **e MWh** ^**−1**^ **)**						
Black carbon	8.5	440	600	300	280	380
Biogenic CO_2_	220	190	240	190	230	200
Supply chain greenhouse gases	42	160	77	62	31	89
Carbon monoxide	0.69	76	36	58	27	55
NMVOC	0.13	49	9.2	31	2.1	29
Total warming	270	920	960	650	560	760
**Net climate impacts per unit of heat output (kg CO**_**2**_ **e MWh** ^**−1**^ **)**						
Net total	69	240	670	300	310	340
**Annual totals (1000 t CO**_**2**_ **e year** ^**−1**^ **)**						
Total cooling	−470	−800	−180	−550	−170	−1700
Total warming	630	1100	590	1000	370	3100
Net total	160	290	410	490	200	1400
Net total as share of Norway GHG	0.3%	0.5%	0.8%	0.9%	0.4%	3%

Significant biogenic CO_2_ warming and albedo cooling are common for all heating alternatives (though with variations): Biogenic CO_2_ is responsible for 27% and 81% of total warming while albedo cooling offsets 27% and 64% of total warming for average wood stove and district heating bioenergy, respectively. For heating stoves, there is an additional important warming effect from BC (constituting 50% of total warming for stoves) and cooling effect from OC (offsetting 26% of total warming). Besides warming from BC and biogenic CO_2_, 15% and 12% of total warming for wood stoves and district heating, respectively, are ascribed to supply chain GHGs, which include methane (CH_4_) and nitrous oxide (N_2_O) emitted directly from stoves or combustion plants. Direct CH_4_ and N_2_O emissions represent, respectively, 50% and 6% of the total supply chain GHG emissions for wood stoves. The most important source of fossil CO_2_ is transport of firewood to the (stove) users, followed by firewood chopping (before retail).

In Fig. 3, the variation in BC and OC impacts across wood stove categories can be a challenge to interpret, as they depend on both overall particulate matter (PM) emissions and the shares of PM that are BC or OC, and because BC and OC have opposing (warming/cooling) effects. Nominal load stoves have much lower PM emissions than partial load stoves, and new stoves have much lower PM emissions than old stoves. BC fractions of PM are appreciably higher for nominal operation than for partial operation, while OC fractions are comparable (Supplementary Table S2). The substantial BC warming and OC cooling associated with partial load stoves is a result of large overall PM emissions. The substantial BC warming for nominal load stoves (especially old stoves) is attributable to high BC fractions of PM for nominal load stoves. These are also the reasons why, in Fig. 3, the net total climate impact is significantly lower for partial load old stoves than for nominal load old stoves.

**Figure 4:**
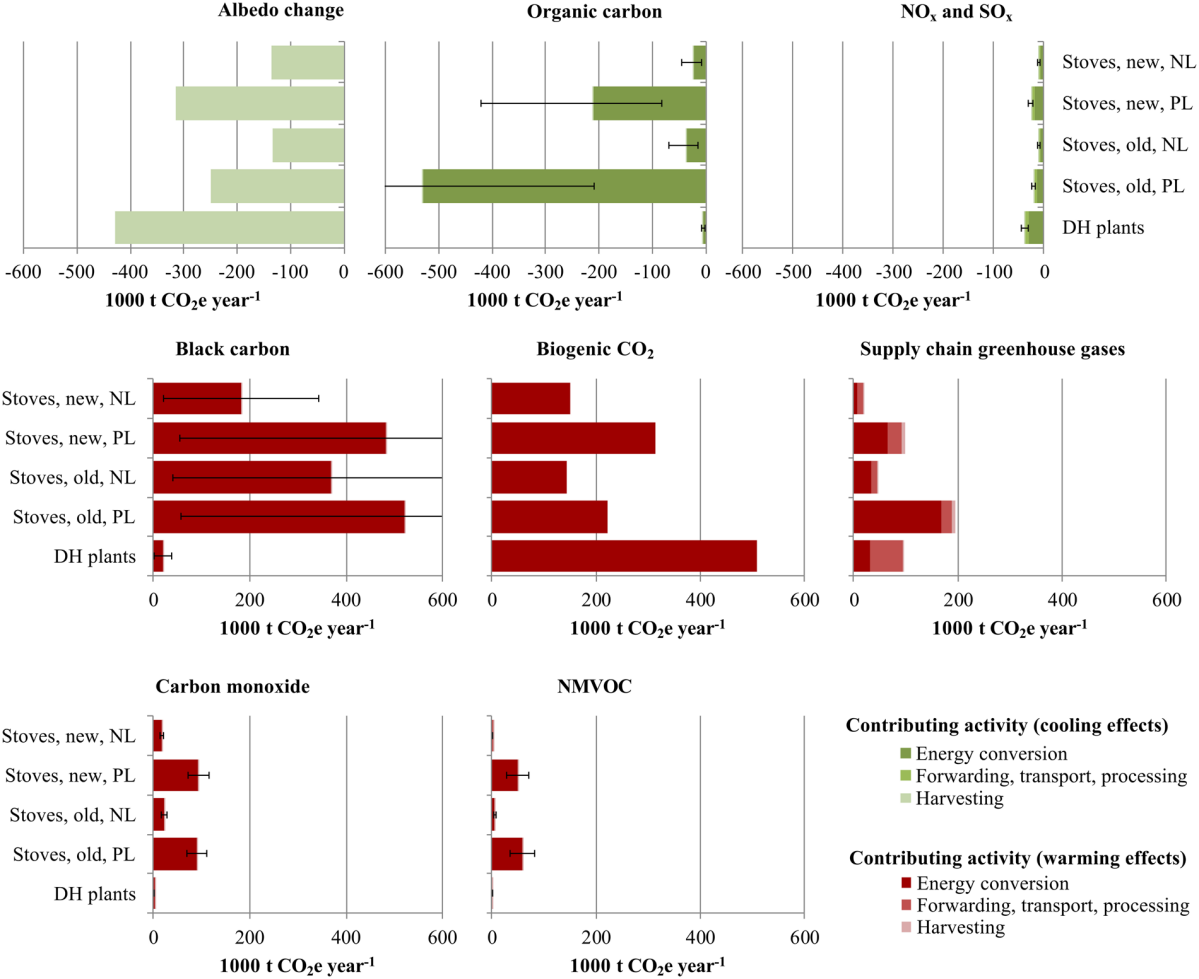
Annual climate impacts of bioenergy from wood stove and district heating. Albedo impacts are attributed to the harvesting activity category. Supply chain GHGs include direct CH_4_ emissions from biomass combustion and storage, besides all fossil GHG. DH = district heating; PL = partial load; NL = nominal load. Ranges reflect uncertainty (±1 standard deviation) for GWP values for NTCFs^10,30^. Range for organic carbon, stoves, old, PL extends to −1100; for black carbon, stoves, new, PL to 910; for black carbon, stoves, old, NL to 700; and for black carbon, stoves, old, PL to 990 (all numbers in 1000 t CO_2_e year^−1^).

Figure 4 shows the annual climate impacts of bioenergy from wood stove and district heating in Norway. Unlike in Fig. 3, impacts are measured as annual absolutes; hence, they depend on heat delivery and harvest volumes (cf. Table 1). The total warming effect amounts to 3.7 Mt CO_2_e year^−1^ and total cooling −2.2 Mt CO_2_e year^−1^, which yields a net total warming of 1.5 Mt CO_2_e year^−1^ (equivalent to 5% of total Norway GHG emissions, as discussed in supplementary Climate warming and cooling effects). As can be seen from Fig. 4, partial load stoves are the dominant cause of climate impacts associated with OC and BC, and of the less important impacts associated with CO and NMVOC. Also evident from the figure is the dominance of energy conversion (i.e., biomass combustion) and harvest activities in causing climate impacts. As an indication of uncertainty stemming from GWP values for NTCFs, ranges (±standard deviation) are included for NTCF results in Fig. 4 based on refs^10,30^.

Our results further substantiate the conclusion of ref.^37^ that BC, despite frequently overlooked in climate impact analysis of bioenergy systems, can be a major climate warming species for small-scale combustion units such as wood stoves. Combustion plants and district heating for providing residential space heating shows lower PM emissions than wood stove heating, owing to higher efficiencies and pollution control equipment installed in combustion plants. Future research and technological improvements can target abatement of BC emissions from wood stoves in order to yield climate benefits.

### County contributions and variations for carbon and albedo impacts

**Figure 5:**
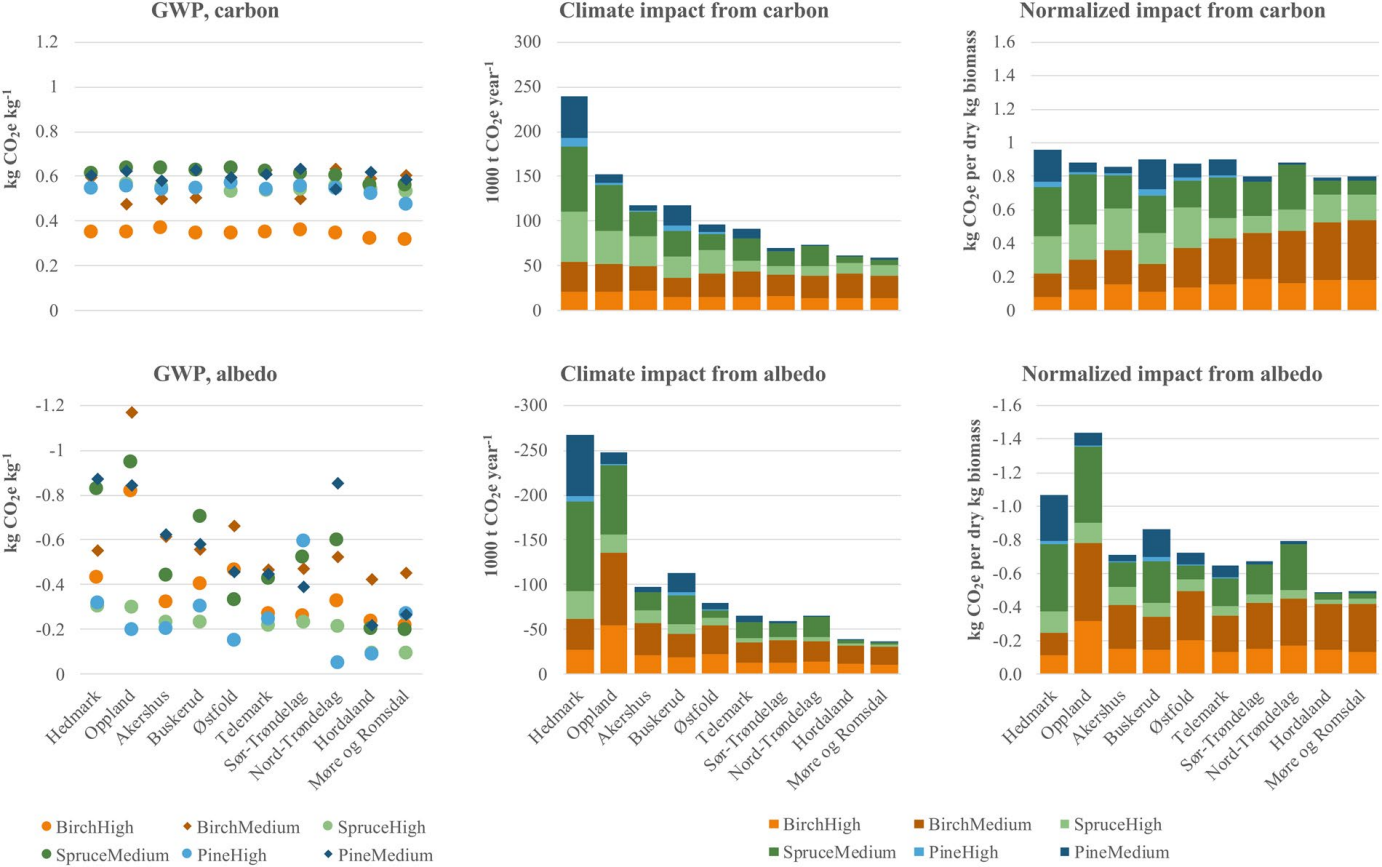
Carbon and albedo global warming potentials (GWPs; left column), annual carbon and albedo climate impacts (centre column) and annual carbon and albedo climate impacts normalized to total annual harvest (right column) by county. Top ten counties in terms of total attributed harvest are shown, with breakdowns into tree species and forest productivity classes (as in Fig. 1). GWP values are weighted averages for two forest classes distinguished by distance to forest road (see Methods).

Albedo GWPs exhibit a much greater scatter than carbon GWPs (Fig. 5). This is evident both across species-productivity classes for single counties and across counties for single species-productivity classes, and arises because snow cover and forest density differences among counties significantly influence albedo GWP. Counties with high attributed harvest levels (cf. Fig. 1) tend to display relatively high albedo GWP (Fig. 5, left column), which amplifies the importance of the highest-harvest counties for albedo climate impacts. When albedo impacts are normalized to total county-level attributed harvest, impacts vary by up to a factor of three among counties (Fig. 5, right column). Meanwhile, normalized climate impacts from carbon vary only slightly, with variations caused mainly by different shares held by birch to total attributed harvest and lower GWP values for birch.

### Limitations and uncertainties

Uncertainties are present in various parts of our analysis, including the mapping of bioresources, compilation of emission inventories, and climate impact characterization. One notable source of uncertainty is the allocation of forest harvest to counties in combination with differences in albedo impact intensities between counties, as discussed in the previous section. Further, the GWP metrics for NTCFs considered are representative of global average responses^10^, although impacts from NTCFs can be spatially and temporally heterogeneous. For instance, the climate impact response to an emission pulse is generally higher in the northern hemisphere, and higher over land than oceans^38^. This particularly applies to aerosol species, owing to their short lifetimes and low degree of mixing in the atmosphere^11^. Regional specific responses and emission metrics for NTCFs are available^39,40^, but additional studies are required to determine their robustness^10,41^. The standard deviation results included in Fig. 4 demonstrate high uncertainty of BC and OC impact estimates for wood stove bioenergy.

Radiative forcing impacts and associated GWP metrics are evaluated under a constant background climate and in the absence of potentially important biophysical feedbacks, such as surface roughness or evapotranspiration changes^10^. Future changes in climate can have two contrasting effects on emission metrics for forest bioenergy in Norway. On the one hand, carbon metrics may decrease as boreal areas experience more favourable growing conditions^34^. On the other hand, albedo metrics may decrease owing to reduced snowfall. Reduced surface albedo in a forest productive region of Norway in March 2100 is estimated to be about 10% or 25% in two trajectories of climate change^42^ (the representative concentration pathways 4.5 and 8.5)^43^. Further, future growth in CO_2_ atmospheric concentrations will increase the fraction of CO_2_ remaining airborne over time because of saturation in ocean and land carbon stocks^44^, and, at the same time decrease the marginal radiative efficiency of CO_2_^10^. However, emission metrics are relatively insensitive to these effects because they are nearly offsetting each other^45,46^.

Another source of uncertainty derives from the emission factor estimations for BC and OC for wood stoves. Uncertainties arise due to a number of factors, including particle concentration variations, sampling and analysis methods, and experimental setup^47^. We expect overall uncertainty to be higher for BC than for OC. For BC, uncertainty is probably higher for new stoves than for old stoves. In the future, emissions of climate-cooling OC are expected to decrease significantly, due to improved combustion technology and replacement of old stoves by new. Emissions of climate-warming BC are more difficult to reduce, as BC formation is more coupled to combustion conditions that are difficult to control or design. A key factor is to avoid local flame extinction at relatively cold locations in the combustion chamber where soot cannot be burnt out. High uncertainties of PM, BC and OC emission factors for residential wood combustion are not unique to our estimates, but are also present in other estimates in literature^37,48^.

The 100-year GWP has been the metric of choice within the UNFCCC to date. Alternative metrics (e.g., 20-year GWP or the 100-year global temperature potential) are conceptually different or incorporate temporal profiles of radiative forcings in different ways^10^. The 100-year GWP is the recommended metric by the UNEP-SETAC guidelines for LCA for addressing “the shorter-term rate of warming (next decades)” (page 63 in ref.^30^).

### Final remarks

Our analysis illustrates the complexity of assessing the climate impacts of forest bioenergy. Factors of complexity include the sizeable differences not only in overall PM emissions among wood stove classes, but also in the fractions of PM that are cooling or warming. The strong location-dependence of normalized albedo impacts represents another element of complexity. The notable and comparable magnitudes of climate cooling and warming effects from diverse forcing agents, the variation in impacts between bioenergy technologies for some forcing agents and the variation across counties for some agents, emphasize the need to consider multiple forcing agents in climate impact analysis of forest bioenergy and provide a rationale for further research in this vein. Unlike non-combustion renewable energy options, forest bioenergy depends on continuous harvest and combustion. While fossil GHG emissions associated with producing infrastructure such as wind turbines will decrease towards zero as economies phase out fossil fuels^49–51^, wood harvest and combustion will continue to cause changes in surface albedo and emissions. The balance of climate cooling and warming effects of bioenergy is hence likely to be a subject of continuous interest.

## Methods

### Wood consumption, efficiencies and emission factors for residential wood stoves

We define four combinations of old/new stoves and partial/nominal load operation, distinguished by different efficiency and emission factor values. We classify stoves produced after 1998 (when new regulations setting limits on particle emissions from stoves were enacted) as new and stoves produced before that year as old. Old stoves typically have glass windows with air flushing, but no insulation of the combustion chamber and no secondary air. New stoves are characterized by advanced technology facilitating clean burning (i.e., secondary air, glass flushing, insulated combustion chamber and sometimes double glasses). In addition, a limited number of ultra-modern stoves also use automatic regulation systems and specially designed combustion chambers to achieve even lower emissions of unburned substances.

We use a weighted average approach to establish wood consumption and emission factors for each of the four stove categories, as briefly described below (see Supplementary Methods and ref.^47^ for further details). Supplementary Table S2 contains the efficiency and emission factor data. We assume that 80% of firewood is birch and other hardwoods and 20% spruce, and other softwoods, based on consumer survey results^52,53^ (see also Methods, Assumptions to overcome data deficiencies for firewood). The relative shares burned in old stoves is 47% and in new stoves 48% (wood burned in open fireplaces represents the remainder of the total, but is not explicitly represented in our analysis)^54^. We assume a distinct use pattern (no night-time burning) for the portion of wood burned in “large” cities (amounting to 6.3% of total firewood)^47^. The respective shares of operating time on partial load and nominal load are derived from the cumulative normal distribution used in the Norwegian test standard^55^: 65% and 35% for old stoves, and 70% and 30% for new stoves. Old stoves are assumed to be operated more often on nominal load than new stoves since they are mostly installed in older, less insulated and leakier houses.

Following the approach outlined above, we determine new emission factors for CO_2_, CH_4_, CO, NMVOC, particulate matter (PM) indicators (including BC and OC) and SO_2_. The factors for CO_2_ take into account the carbon content of non-CO_2_ emission components. Emission factors for N_2_O and NO_x_ are the same as in the Norwegian emission inventory method^56^. We here use black carbon (BC) as synonymous to elemental carbon (EC), although they are defined differently (BC refers to optical properties, EC to physical properties^57^). It may be noted that to a larger degree than in the rest of Europe, wood stoves in Norway are fired with limited combustion air supply (i.e., operation at below the stoves’ nominal power), contributing to more unfavourable combustion conditions and higher emissions of unburned substances for Norwegian stoves^58,59^.

Based on experimental data from past tests^47^, we assume efficiencies for old stoves of 67% and 65% at part and nominal load, respectively, higher than the 50% currently assumed by Statistics Norway^56^. For new stoves at part and nominal load, we assume 72% and 69%, in line with the 70% assumed by Statistics Norway.

### Biomass consumption, efficiencies and emission factors for combustion plants

We collect information on type of biomass fuel, thermal capacity and efficiency for about 600 biomass district heating plants from an official operation permits database^60^ and direct communication with Enova SF under the Norwegian Ministry of Petroleum and Energy. Only district heating plants are considered here, excluding localized heat generation not connected to a heat distribution network (e.g., combustion facilities delivering heat for internal use in pulp and paper or sawmilling industries). We classify the plants into five heat output capacity categories (cf. Table 1) and five main biomass fuel type categories (wood chips, sawdust/shavings, pellets, briquettes and waste wood; some less important fuel types, for instance husk, are not explicitly represented). Based on the combustion plant survey results, we assume that efficiency increase with plant size (from 82–83% for the smallest size category to 93–95% for the biggest), with some variations depending on the fuel type. We assume emission factor values by evaluating information from multiple sources^56,61–63^. The fractions of PM_10_ that are BC and OC are assumed to be 4.3% and 17%, respectively^64^. See Supplementary Methods for further details and Supplementary Table S3 for numerical efficiency and emission factor data.

### Supply chain operations and associated emissions

We employ the method of life cycle assessment (LCA) to attribute in a coherent manner emissions occurring over product lifespans and supply chains to the bioenergy products under study^19^. In our analysis, supply chain operations comprise any pre-harvest activities (seedlings production and planting, forest thinning), operations that take place in the forest (e.g., sawing, chopping, loading, forwarding), fuel storage and processing (e.g., chipping), and transport by car (for firewood) or pipeline (district heating) to final users. We assume 10% overall heat loss in district heating pipes^65^. In order to determine GHG and NTCF emissions associated with fossil fuel combustion, we collect information on key parameters describing forests and wood harvest in Norway (e.g., mean stem size, area of cutting sites, tree species distribution) from the Norwegian national forest inventory^66^, and use this information to group forest area in Norway into 36 cases according to dominating species, forest productivity and region. Next, we employ cost productivity models (most notably, from refs^67–72^) to establish detailed accounts of productive machine hours for each stratum, and then estimate the associated diesel fuel consumption and emissions using conversion factors from refs^61,73^. Finally, inventories of equipment, material and energy requirements are connected to the LCA database Ecoinvent^36^ in order to achieve extensive coverage of effects indirectly associated with bioenergy. Production of key infrastructure and assets (shed, tractor, power saw, wood stove, combustion facility, etc.) is taken into account.

Additional details are added to the supply chain analysis for biogenic CO_2_ and albedo change specifically. Unlike the employed GWP values for GHGs and NTCFs, the employed GWPs for CO_2_ and albedo are explicit in location (aggregated at a county level), tree species and forest productivity class. Hence, an analysis explicitly connecting biomass burned and biomass harvest by counties, species and productivity classes becomes necessary. We achieve this by collating and balancing data from surveys of wood harvest and produced biomass products^66^, firewood consumption by counties^52^, and type and amount of fuel consumption of district heating plants (based on survey undertaken in this study). Biomass is burned in industry for the production of bioenergy fuels (i.e., woodchips, pellets, briquettes) or for the production of intermediate products (e.g., sawdust) used to produce bioenergy fuels. Drawing upon ref.^74^, we assign forest biomass burning in sawmill and fuel-producing industries to district heating bioenergy using mass-based allocation. Consequently, quantities of wood harvest attributed to district heating bioenergy in our analysis exceed the quantities of fuel combusted in the district heating plants by roughly 15%.

### Assumptions to overcome data deficiencies for firewood

Estimates of Norwegian household firewood consumption in 2010 by counties are available from Statistics Norway, based on consumer survey results^52,53^. According to the survey results, 40% of the total consumption is sourced from commercial suppliers, implying that 60% comes from private (non-commercial) sources. Firewood trade statistics indicate that firewood import to Norway constituted 10–16% of total consumption in 2000–2016, and that firewood export from Norway is relatively small (at least one order of magnitude smaller than import)^52^. In addition, a portion of the commercial firewood consumption originates from recovered wood (e.g., from surplus wood from timber production) that cannot easily be traced via forestry statistics. Owing to these factors, using information from the Norwegian national forest inventory-based harvest model^75^ we are able to cover only a small portion (7%) of total firewood consumption. For the remainder consumption, we lack county-specific information on forest parameters and harvest. We apply the following assumptions and simplifications to overcome this data gap problem. One, we treat all firewood production as if it was produced in Norway (i.e, disregarding potentially different production for net imported wood). Two, we assume that the county distribution of firewood production mirrors the county distribution of consumption as indicated by consumer survey results^52^. Three, we implement a fixed share of 80% birch and other hardwoods and 20% spruce and other softwoods across all counties. This is in rough correspondence with statistics on commercial firewood production from timber in Norway in 1996–2005^52^ (more recent data or data pertaining to non-commercial firewood production are not available). Four, we uniformly adopt county-specific ratios between spruce and pine harvest, between different forest site classes and between different distances to forest road from the Norwegian national forest inventory-based harvest model^75^, assuming these ratios are representative for all firewood harvest.

### Post-harvest carbon and albedo dynamics

Stand-replacing forest disturbances alter the net exchange of CO_2_ between the land and atmosphere, with consequences for CO_2_ atmospheric concentration and climate system^76,77^. In addition to CO_2_ emissions from oxidation of harvested products, post-harvest forest stands are usually a source of carbon for some years after disturbance because CO_2_ emissions from heterotrophic respiration (Rh) exceed carbon sequestration in new trees via net primary productivity (NPP)^78,79^. Once residues have decomposed and NPP increases, the net ecosystem exchange (NEE, NEE = Rh – NPP) becomes negative, and the forest ecosystem acts as a net carbon sink. The transition from carbon source to carbon sink usually occurs within the first two or three decades following stand replacement, and largely depends on the amount and decay rate of post-harvest forest residues remaining in the forest to decompose^80^.

Post-harvest NPP is determined using an empirical harvest model based on the Norwegian national forest inventory, which provides spatial explicit information on tree species relative abundancy, volume, age, site-class, and distance to forest road of the forest plot^75^. The most abundant tree species in Norwegian forests are spruce (*Picea Abies*), pine (*Pinus Sylvestris*) and birch (mixed species). The Rh response to the harvest event is obtained from YASSO07, which estimates changes in soil carbon and decomposition rate of forest harvest residues with climate-explicit variables such as the mean annual temperature, average annual precipitation, and mean amplitude of average monthly minimum and maximum temperature^81^. Post-harvest net CO_2_ exchanges are estimated for each plot after combining the NPP and Rh profiles. Results are then aggregated at a county level per dominant tree species (spruce, pine, and birch), distance from forest road (short or medium), and site productivity class (medium or high). We therefore have 12 possible combinations of NEE profile per county. Because forest distribution in the country is heterogeneous, there are missing data in some counties for certain tree species. For instance, forests at high latitude are little productive, and therefore left out from our analysis.

Changes in surface albedo occur after forest harvest, when the solar reflective property of the surface is perturbed and the surface masking effects of trees is reduced^82^. Open land usually has higher albedo (i.e., higher reflectivity of incoming solar radiation) than forested land, and the difference is amplified in regions affected by seasonal snow cover^12,15^. When the forest regrows the surface albedo change gradually declines and returns to the pre-harvest level. This temporary perturbation causes a cooling contribution that can be of the same order of magnitude of the impacts associated with carbon fluxes^24,83^. We use post-harvest county-average volume increments to predict monthly-mean albedo dynamics using a simultaneous unmixing and non-linear regression model^84^. The model is based on multi-year satellite retrievals of MODIS surface albedo (MCD43A3)^85^, high resolution land cover maps^86^, and meteorological records^87^ and uses forest structure information (volume and age) and climate variables (temperature and snow water equivalents) to characterize albedo variations in Norway across latitude, seasons, land cover types, and topography. It relies on an extensive analysis of surface albedo changes across space and in time in Norwegian forests^88^.

### Climate impact assessment

NEE profiles represent the ecosystem carbon response to the harvest event and are the basis for the computation of emission metrics for forest bioenergy. These metrics are computed following the standard protocol used by the 5th IPCC Assessment Report^10^, which uses a multi-model mean for the impulse response function (IRF) of CO_2_^44^. First, the IRF associated with CO_2_ emissions from biomass combustion are produced for each case (county, tree species, site index, and distance to forest road) after the integration with the global carbon cycle through a mathematical convolution^34^. The IRF describes the case-specific change in CO_2_ atmospheric concentration from bioenergy CO_2_ emissions and the associated site-specific NEE profiles. This is then converted into radiative forcing as the product between the specific IRF for CO_2_ from bioenergy and CO_2_ radiative efficiency (defined as the radiative forcing per kg increase in atmospheric burden of the gas), under the common assumption that for sufficiently small emissions and approximately constant background conditions the radiative efficiency can be approximated as time-invariant^89^. According to the latest IPCC assessment report, the background atmospheric CO_2_ concentration is held constant to the average concentration in 2010 (389 ppmv). By integrating the RF we then obtain the AGWP which is then used to calculate the GWP metric with a time horizon of 100 years^10^.

Radiative forcing from changes in surface albedo are computed according to a simplified 1-layer atmospheric transfer model^24,25^, which is found to perform reasonably well when compared with more sophisticated models^90^. A change in albedo is translated to a change in global radiative forcing at the top of the atmosphere after combination with the monthly-mean incoming solar radiation at the surface and a global average atmospheric transmittance parameter. Spatial explicit mean incoming solar radiation is gathered per month and latitudinal/longitudinal degree for all Norway as the 22-year mean of the downwelling solar radiation flux at surface level^91^. The radiative forcing is then normalized to the unit of biomass harvested, time-integrated, and used to compute GWP with a time horizon of 100 years.

The present calculated GWP values (Fig. 2) are generally in line with those reported in past studies. A study that quantifies spatially explicit CO_2_ emission metrics for forest bioenergy estimates mean 100-year GWPs for Norway between 0.44 and 0.69 kg CO_2_e per kg CO_2_ (national aggregated value covering birch, spruce and pine species)^34^. Another study computes 100-year GWPs for a Norwegian spruce plantation in the county of Hedmark in the range of 0.44 and 0.62 kg CO_2_e per kg CO_2_^92^. Previous estimates of 100-year GWP for surface albedo from spruce plantations in Hedmark are between −0.3 and −0.4 kg CO_2_e per kg CO_2_^26,31^.

### Data availability

The data that support the findings of this study are available from the corresponding authors upon request.

## Electronic supplementary material

Supplementary information
