# Supplementary material for: Cooling aerosols and changes in albedo counteract warming from CO2 and black carbon from forest bioenergy in Norway
Source: Sci Rep. 2018 Feb 19;8:3299. doi: 10.1038/s41598-018-21559-8 (PMC5818662; doi:10.1038/s41598-018-21559-8)
Supplement: Supplementary file 1 — Supplementary information [file 41598_2018_21559_MOESM1_ESM.docx]

**Supplementary information:**

**Cooling aerosols and changes in albedo counteract warming from CO_2_ and black carbon from forest bioenergy in Norway**

Anders Arvesen^1,*^, Francesco Cherubini^1,*^, Gonzalo del Alamo Serrano^2^, Rasmus Astrup^3^, Michael Becidan^2^, Helmer Belbo^3^, Franziska Goile^2^, Tuva Grytli^1^, Geoffrey Guest^1,4^, Carine Lausselet^1^, Per Kristian Rørstad^5^, Line Rydså^2^, Morten Seljeskog^2^, Øyvind Skreiberg^2^, Sajith Vezhapparambu^1^, Anders Hammer Strømman^1^

^1^ Industrial Ecology Programme and Department of Energy and Process Engineering, Norwegian University of Science and Technology (NTNU), Norway

^2^ SINTEF Energy Research, Trondheim, Norway

^3^ Norwegian Institute of Bioeconomy Research, Norway

^4^ National Research Council Canada, Canada

^5^ Norwegian University of Life Sciences, Norway

^*^ Email: anders.arvesen@ntnu.no (A.A.); francesco.cherubini@ntnu.no (F.C.).

#

# Global warming potentials (GWPs)

Fig. 2 in the main article shows the GWP values for CO_2_ emissions and post-harvest albedo dynamics for birch and spruce. Corresponding results for pine are shown in Supplementary Fig. S1. Supplementary Fig. S2 shows net GWP (combined CO_2_ and albedo) associated with birch, spruce and pine.

| 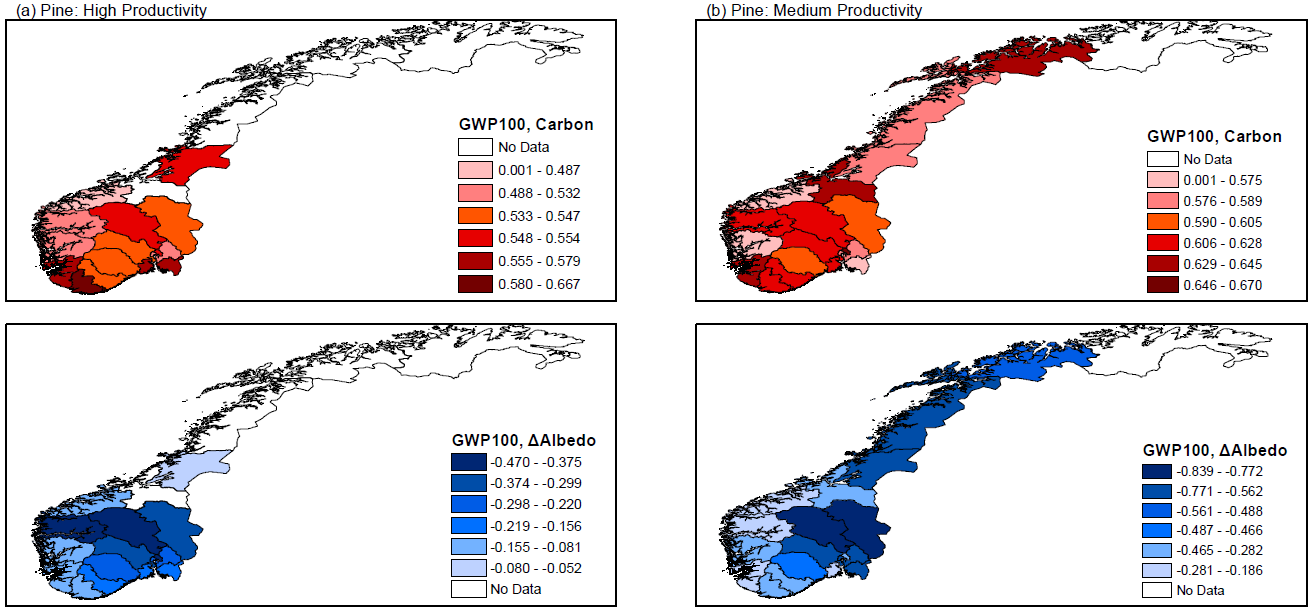 |
| --- |

**Supplementary Figure S1. Global warming potentials calculated with a 100-year time horizon (GWP100) for CO_2_ emissions and changes in surface albedo associated with pine forest bioenergy in Norway.** Plots in left and right columns are for high and medium forest productivity classes, respectively. GWP values are expressed in kg CO_2_e per kg of CO_2_ emissions from biomass combustion. The maps were created by using ArcMap 10.3 software (http://desktop.arcgis.com/en/arcmap/).

| 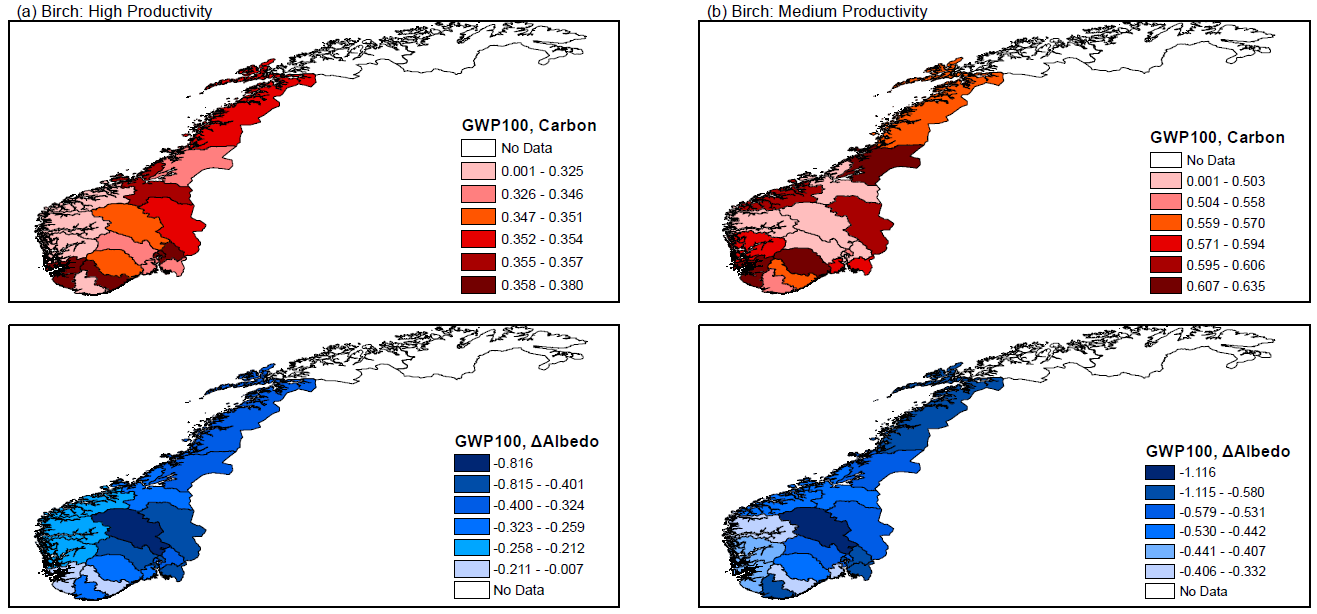  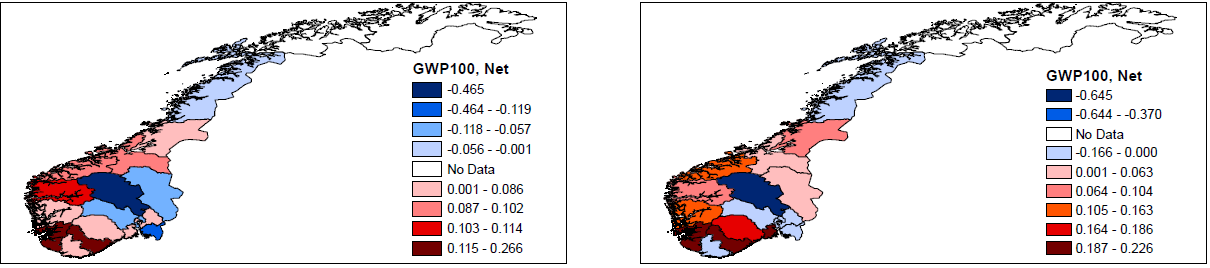 |
| --- |
| 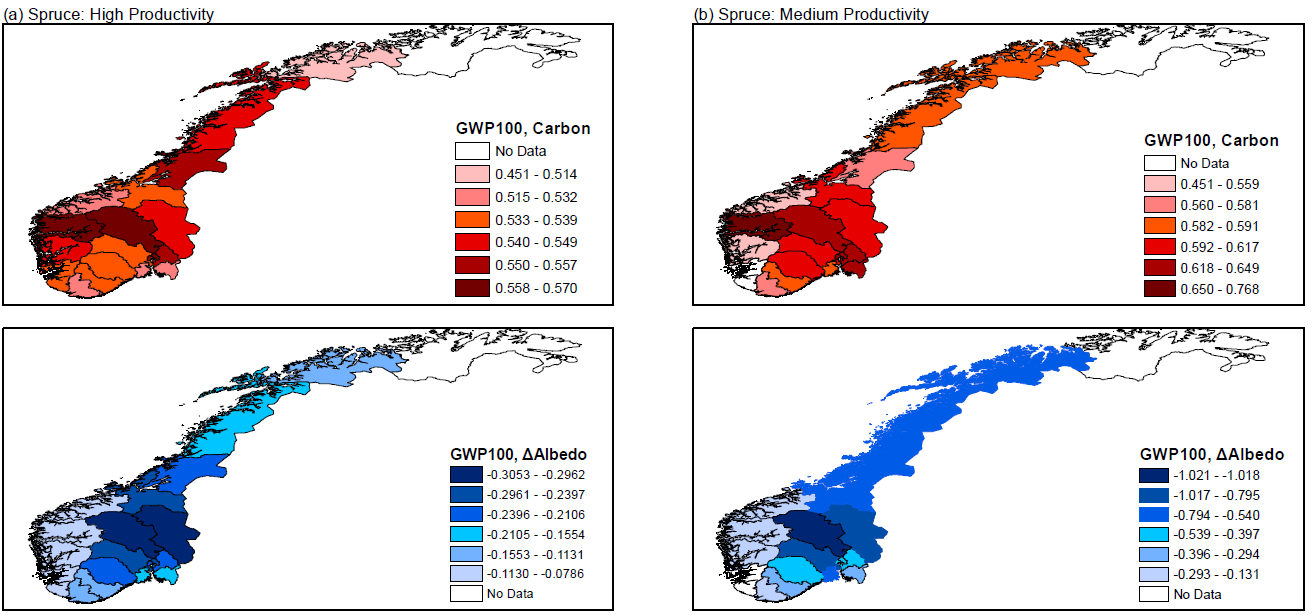 |
| 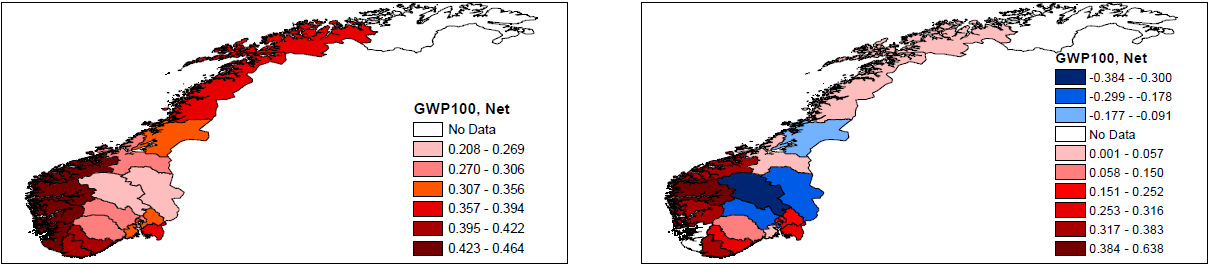 |
| 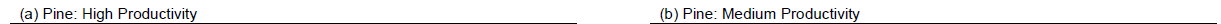  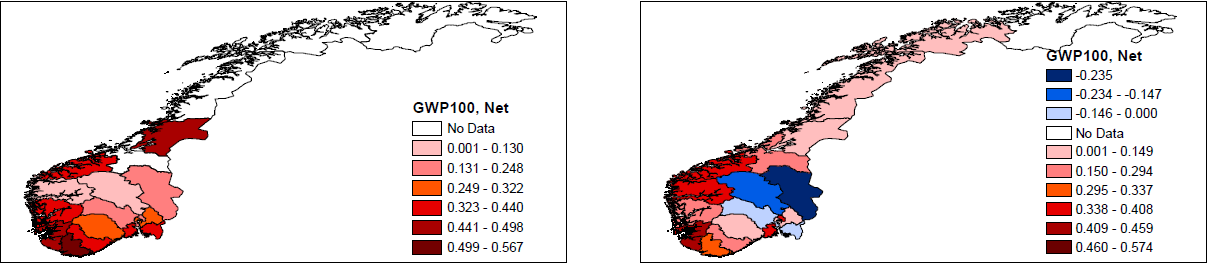 |

**Supplementary Figure S2. Net global warming potentials calculated with a 100-year time horizon (GWP100) associated with birch, spruce and pine forest bioenergy in Norway.** Net GWPs are combine contributions from CO_2_ and albedo. Plots in left and right columns are for high and medium forest productivity classes, respectively. GWP values are expressed in kg CO_2_e per kg of CO_2_ emissions from biomass combustion. The maps were created by using ArcMap 10.3 software (http://desktop.arcgis.com/en/arcmap/).

# Climate warming and cooling effects

Supplementary Fig. S3 provides an alternative (aggregated) display of Fig. 4 in the main article, showing annual climate impacts of wood stove and district heating bioenergy in Norway.

**Supplementary Figure S3. Annual climate impacts of wood stove and district heating bioenergy.** Albedo impacts are subsumed under the harvesting activity category. DH = district heating; PL = partial load; NL = nominal load.

**Supplementary Table S1. Annual** climate cooling and warming effects by climate forcers and technologies, shown as absolute totals and as percentage shares of total GWP100 in Norway. Norway total GHG is 53.9 million tonnes CO_2_e in 2015^1^. This Norway total ignores biogenic CO_2_, albedo and NTCFs. PL = partial load; NL = nominal load.

|  | **District heating** | **Stoves, old, PL** | **Stoves, old, NL** | **Stoves, new, PL** | **Stoves, new, NL** | **Stoves, combined** | **All, combined** |
| --- | --- | --- | --- | --- | --- | --- | --- |
|  |  |  |  |  |  |  |  |
| **Annual totals as share of Norway GHG** | |  |  |  |  |  |  |
| Total cooling | -0.9 % | -1 % | -0.3 % | -1 % | -0.3 % | -3 % | -7.2 % |
| Total warming | 1 % | 2 % | 1 % | 2 % | 0.7 % | 6 % | 13 % |
| Net total | 0.3 % | 0.5 % | 0.8 % | 0.9 % | 0.4 % | 3 % | 5.4 % |

Supplementary Table S1 indicates the total climate effects of bioenergy as percentage shares of nationwide Norway GHG emissions. Looking at wood stove bioenergy, total climate warming and cooling effects are each more than one order of magnitude smaller than the total GHG emissions for Norway. The net total impacts of stoves amount to 3% (6% for warming less 3% for cooling) of Norway GHG. Firewood use in our analysis corresponds to 9% of total household final energy consumption (including fuel consumption in private cars) in 2013^1^. We note that unlike the current climate impact estimates for bioenergy, the GHG figures for Norway do not consider biogenic CO_2_, albedo or NTCFs. Norway’s pledge (“Intended Nationally Determined Contribution”) under the UNFCCC Paris Agreement is to reduce GHG by at least 40% by 2030 relative to 1990^2^, equivalent to about 42% relative to 2015.

The total amount of particulate matter (PM) emissions from stoves in our results is 18 000 tonnes (Supplementary Fig. S4), equivalent to about one third of reported total PM emissions in Norway (about 55 000 tonnes per year in 2013-2015^1^). PM emissions from stoves is a cause for (local and time-specific) concerns about reduced air quality, but this is outside the scope of the current article, which focuses on the climate-altering organic carbon and black carbon^3^ fractions of PM.

**Supplementary Figure S4. Total annual emissions of particulate matter (PM) (*left axis*) and total emissions of PM per unit heat output (*right axis*) for wood stove and district heating bioenergy.**

Besides the factors already discussed in the main article, it may be noted that climate warming associated with CO and non-methane VOC (NMVOC) are noticeable but comparatively small for wood stoves, and insignificant for district heating (Figs 3 and 4 in main article). The small magnitudes of NOx and SOx cooling (Figs 3 and 4 in main article) are not surprising given that NOx and SOx emissions from bioenergy are known to be very small in comparison to NOx and SOx from transport and oil and gas extraction in Norway^1^. SOx emissions attributed to bioenergy in our results are largely connected to supply chain fossil fuel use.

# Methods

**Wood consumption, efficiencies and emission factors for residential wood stoves.** Emission studies for wood stoves typically reflect specific local/regional characteristics and therefore are not straightforward applicable to other countries. As is noted in Methods in the main article, wood heating characteristics in Europe differs significantly from country to country. Both heating patterns and technologies in use vary due to differences such as climate, type of buildings, access to resources, type of basic heating and firing traditions^4, 5^. Further, several emission measurement test methods are in use due to the lack of a common approval test method, resulting in major differences in reported emission factors^6^.

Roughly speaking, wood stove technology can be related to a stove’s year of production. Wood stoves prior to 1970-80 in Norway had no or almost no glass windows, no wall air flushing, no insulation of combustion chamber and no secondary air. Stoves produced between 1970-80 and up to 1998 are categorized as old stoves in our assessment. Such stoves typically had glass windows with air flushing but no insulation of the combustion chamber and no secondary air. Almost all stoves produced after 1998 (when new regulations setting limits on particle emissions from stoves were enacted) exhibit what we call new technology, i.e. secondary air, glass flushing, insulated combustion chamber and sometimes double glasses. In addition, a few ultramodern stoves also uses automatic regulation systems and specially designed combustion chambers to achieve even lower emissions of unburnt. Supplementary Fig. S4 illustrates main differences between old and new wood stove technology.


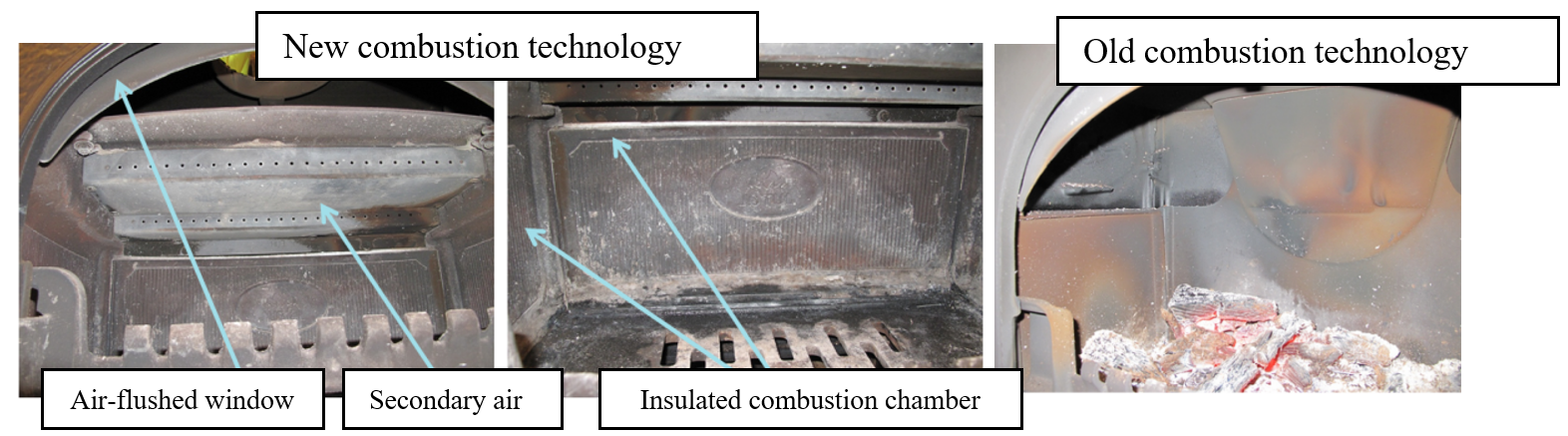


**Supplementary Figure S5. Illustration showing main differences between old and new wood stove technology in Norway.**

As briefly explained in Methods in the main article, we use a weighted average approach to establish wood consumption and emission factors for each of the four stove categories. The work involved collecting and evaluating in-house experimental data from various stoves tested by SINTEF dating back several decades as well as reviewing external/internal literature/reports to confirm whether or not existing Norwegian emission factors were in need of revision. Since a stove’s load has a significant influence on its performance in terms of emissions of unburnt, emission factors were collected for nominal and part load for both old and new wood stoves. Wood stove efficiencies where evaluated in the same manner. In addition, the other current emission factors were evaluated, and updated ones were suggested where better values than the existing ones used were found based on literature data or wood composition analyses (as max values based on element concentration in the wood).

Taking the Norwegian Emission Inventory 2013^7^ as a starting point, new emission factors have been derived where possible and if technology and load sensitive, weighted emission factors have been calculated based on a proposed new method. The weighting according to the new proposed method is based on statistical data from 2013 providing data on firewood consumption in old stoves, new stoves and fireplaces, respectively given as **47.4**, **48.3** and **4.3** % of the total consumption, as well as percent wood consumption in large cities and the rest of the country, respectively given as **6.3** and **93.7** %. The percentwise operating time on part load and nominal load for old and new stoves P_part_/P_nom_, **65/35** and **70/30** % respectively, were derived from the cumulative normal distribution used in the Norwegian test standard^8^ with the assumption that the main part of the Norwegian stoves are class 2 stoves (able to operate below 1.25 kg dry wood per hour but above 0.8 kg/h). The percent operation time, P_nom_, on nominal load was calculated for a value of 1.875 kg/h which is the average between what was defined as nominal and part load, 8 kW (≈ 2.5 kg/h) and 4 kW (≈ 1.25 kg/h), respectively. The percent operation time on part load is then calculated as P_part_ = 1 - P_nom_. Old stoves are assumed to be operated more often on nominal load than new stoves since they are mostly installed in older and hence less insulated and leakier house constructions.

Efficiencies were also evaluated for old and new stove technologies. Measurement data from previous tests showed that the efficiencies currently used by statistics Norway for old stoves, i.e. **50** %, is too low, and should rather be, about **67** and **65** % at part and nominal load, respectively. The current used efficiency of new stoves, i.e. **70** %, is in line with what was found in this work, **72** and **69** % at part and nominal load, respectively. The slightly higher efficiencies for part load than nominal load are explained by lower chimney temperatures, which more than compensates for efficiency reduction due to relatively more unburnt carbon.

Compared to emission factors used in the Norwegian Emission Inventory 2013, emission factors for TSP, PM_10_, PM_2.5_, EC, OC, CO, CH_4_ and NMVOC were revised based on an evaluation of own technology and load representative data, which were weighted according to the proposed method above to arrive at a representative emission factor for each component. Emission factors for SO_2_ were revised based on fuel composition analyses and statistical data for soft- and hardwood consumption in Norway, as well as assuming a reduced SO_2_ emission level for old stoves at part load operation. Emission factors for NOx and N_2_O were not changed due to no new available representative data. Finally, emission factors for CO_2_ were calculated based on the fuel wood carbon content, but correcting for the carbon content in non-CO_2_ emission components.

Supplementary Table S2 contains the efficiency and selected emission factor values for wood stoves in the current work. Employed lower heating value assumptions are 18.7 MJ dry kg^-1^ for birch and other hardwoods and 19.1 MJ dry kg^-1^ for spruce and other softwoods. Birch and other hardwoods constitute 80% and spruce and other hardwoods constitute 20% of firewood consumption (assumption, see Methods in main article).

Supplementary Table S2. Wood stove efficiency (%) and emission factors (g/kg) for the four categories of wood stoves. Emission factor values represent direct emissions only (i.e., excluding supply chain emissions). They are shaded according to their relative magnitude, with red (green) shading denoting big (small) magnitude. The shadings are commensurate across stove categories for a given emission compound, but not commensurate across compounds.

|  | **Old stoves, partial load** | **Old stoves, nominal load** | **New stoves, partial load** | **New stoves, nominal load** |
| --- | --- | --- | --- | --- |
|  |  |  |  |  |
| Efficiency | 67 % | 65 % | 72 % | 69 % |
|  |  |  |  |  |
| **Emission factors (g kg^-1^)** |  |  |  |  |
| Organic carbon | 1.9E+01 | 2.2E+00 | 5.8E+00 | 1.4E+00 |
| Black carbon | 9.6E-01 | 1.2E+00 | 6.8E-01 | 5.9E-01 |
| CH_4_ | 2.1E+01 | 7.3E+00 | 5.3E+00 | 5.3E-01 |
| CO | 1.3E+02 | 5.8E+01 | 1.0E+02 | 4.5E+01 |
| CO_2_ | 1.4E+03 | 1.7E+03 | 1.6E+03 | 1.8E+03 |
| N_2_O | 3.2E-02 | 3.2E-02 | 3.2E-02 | 3.2E-02 |
| NMVOC | 3.1E+01 | 5.5E+00 | 2.1E+01 | 1.3E+00 |
| NO_x_ | 9.7E-01 | 9.7E-01 | 9.7E-01 | 9.7E-01 |
| PM10 | 3.4E+01 | 3.5E+00 | 1.1E+01 | 2.0E+00 |
| SO_2_ | 3.3E-01 | 3.5E-01 | 3.5E-01 | 3.5E-01 |

**Biomass consumption, efficiencies and emission factors for combustion plants.** As is explained in the corresponding Methods subsection in the main article, we rely on an official operation permits database^9^ to assume thermal efficiencies of five size classes of district heating combustion plants. Table 1 in the main article shows the employed assumed efficiency values (the ranges in the efficiency values in the table reflect slight variations depending on the fuel type). Employed lower heating value assumptions vary between 17.6 and 19.0 MJ dry kg^-1^ depending on fuel type, averaging at about 18.7 MJ dry kg^-1^ (weighted average across fuel types).

Emission factor values are own assumptions based on an overall evaluation of various data sources^7, 10, 11, 12^. For combustion plant sizes > 1 MW, assumed emission factor values for dust, CO, NO_x_ and SO_2_ are in compliance with current air pollution regulations in Norway (combustion plant sizes < 1 MW are not subject to regulatory emission limits)^12^. The fractions of PM_10_ that are black carbon and organic carbon are assumed to be 4.3% and 17%, respectively, for all combustion plant size classes^13^. For biogenic CO_2_, we employ a generic emission factor of 1.8 kg CO_2_ dry kg^-1^.

Supplementary Table S3. Combustion plant efficiency (%) and emission factors (g/kg) for the five combustion plant size classes. Emission factor values represent direct emissions only (i.e., excluding supply chain emissions). They are shaded according to their relative magnitude, with red (green) shading denoting big (small) magnitude. The shadings are commensurate across stove categories for a given emission compound, but not commensurate across compounds.

|  | **Bio DH < 1 MW** | **Bio DH 1<10 MW** | **Bio DH 10<20 MW** | **Bio DH 20<50 MW** | **Bio DH >50 MW** |
| --- | --- | --- | --- | --- | --- |
|  |  |  |  |  |  |
| Efficiency | 82-83% | 85-86% | 89-91% | 91-93% | 93-95% |
|  |  |  |  |  |  |
| **Emission factors (g kg^-1^)** |  |  |  |  |  |
| Black carbon | 9.1E-02 | 4.1E-02 | 2.1E-02 | 8.0E-03 | 8.0E-03 |
| Organic carbon | 3.6E-01 | 1.6E-01 | 8.1E-02 | 3.3E-02 | 3.3E-02 |
| CH_4_ | 1.3E-05 | 7.6E-06 | 7.5E-06 | 7.6E-06 | 5.6E-06 |
| CO | 1.1E-02 | 1.9E-03 | 1.9E-03 | 1.3E-03 | 5.1E-04 |
| CO_2_ | 1.8E+03 | 1.8E+03 | 1.8E+03 | 1.8E+03 | 1.8E+03 |
| NO_x_ | 1.7E-03 | 1.3E-03 | 1.3E-03 | 1.3E-03 | 1.3E-03 |
| SO_2_ | 1.6E-04 | 4.5E-05 | 4.5E-05 | 4.5E-05 | 4.5E-05 |

**Supply chain operations and associated emissions.** As is noted in the main manuscript (Methods, Supply chain operation and associated emissions), we allocate the forest area in Norway to 36 strata, according to dominating species, site productivity class and region. Forest parameters like mean stem size, mean size (i.e. total volume) of cutting sites, species distribution, and assortment output distribution are obtained from the Norwegian national forest inventory^14^. Further, biomass supply chains are modeled to estimate the costs and consumption of commodities relating to harvesting, transport and processing of biomass. For example, for virgin forest roundwood assortments, the supply chain includes harvesting, terrain transport, road transport, processing and storage. Each of these steps are modeled individually to estimate specific unit time consumption (PMh_15_ t_d_^-1^), diesel and energy consumption (l t_d_^-1^, kWh t_d_^-1^) and direct emissions for each stratum.

Productivity and fuel consumption models were found in international scientific publications or research reports of Scandinavian origin (most notably, from refs ^15, 16, 17, 18, 19, 20^). The productivity models of ref. ^15^ is used to estimate the specific time consumption for harvesting in the individual stratums. In these models (as in most harvesting productivity models), the productivity is basically governed by mean tree size and species. The fuel consumption of harvesters and forwarders was estimated using ref. ^21^, where the fuel consumption is related to mean tree size and forwarding distance respectively. Supplementary Fig. S5 illustrates the principal approach for estimating costs, consumption of commodities and production for biomass supply for each forest stratum. As a final, additional step, inventories of equipment, material and energy requirements are connected to the LCA database Ecoinvent^22^.


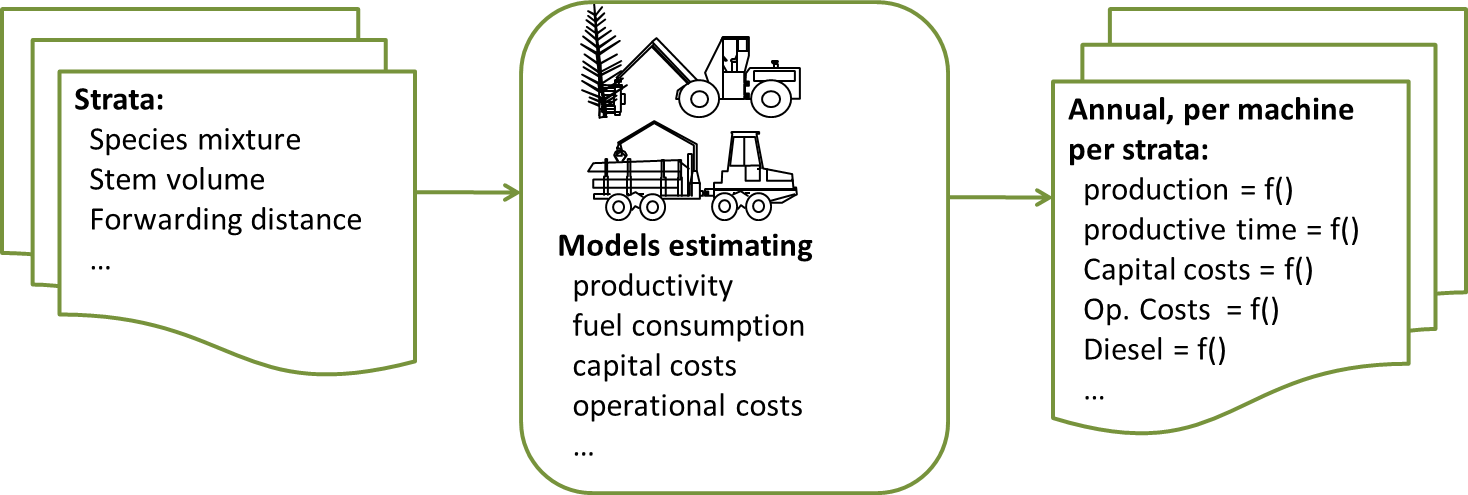


**Supplementary Figure S5**. Illustration of the basic approach for estimating costs, consumption of commodities and production for biomass supply for each forest stratum.

In an additional step, inventories of equipment, material and energy requirements are connected to the LCA database Ecoinvent^22^. In order to estimate the emissions associated with producing wood stoves, we assume wood stoves contains 170 kg cast iron and smaller amounts of other materials. We model the materials production using the Ecoinvent LCA database^22^. The other materials contained in wood stoves are: Soapstone (0 kg per old stove and 20 kg per new stove; as the exact material is missing in Ecoinvent, limestone is used as a proxy); calcium silicate insulating panels (5 kg per old stove and 10 kg per new stove; “fibre cement facing tile” in Ecoinvent is used as a proxy); flat glass (5 kg per old stove and 10 kg per new stove). The assumed lifetime for wood stoves is 20 years. The estimations of emissions associated with all infrastructure and assets other than wood stoves (shed, tractor, power saw, combustion facility, etc.) are based on Ecoinvent^22^.

# References

1. Statistics Norway. Available from: https://www.ssb.no/en/statistikkbanken [Accessed 4 October 2016].

2. KLD. Ny utslippsforpliktelse for 2030 – en felles løsning med EU [New emission reduction pledge for 2030 - a shared solution with the EU]: Ministry of Climate and Environment (KLD), Norway (2015).

3. Myhre G, Shindell D, Bréon F-M, Collins W, Fuglestvedt J, Huang J*, et al.* Anthropogenic and Natural Radiative Forcing. In: Stocker TF, Qin D, Plattner G-K, Tignor M, Allen SK, Boschung J*, et al.* (eds). *Climate Change 2013: The Physical Science Basis. Contribution of Working Group I to the Fifth Assessment Report of the Intergovernmental Panel on Climate Change*. Cambridge University Press: Cambridge, United Kingdom and New York, NY, USA (2013).

4. Karlsvik E. Current firewood firing technology, Quality Wood Project Report 6/2007, Project EIE/06/178/SI2.444403. https://ec.europa.eu/energy/intelligent/projects/en/projects/quality-wood (2017).

5. Alves C, Gonçalves C, Fernandes AP, Tarelho L, Pio C. Fireplace and woodstove fine particle emissions from combustion of western Mediterranean wood types. *Atmospheric Research*, **101**(3)**:** 692-700 (2011).

6. Nussbaumer T, Klippel N, Johansson L. Survey on measurements and emission factors on particulate matter from biomass combustion in IEA contries. *Paper presented at "16th European Biomass Conference and Exhibition"*. Valencia, Spain (2008).

7. Statistics Norway. The Norwegian Emission Inventory 2013, https://www.ssb.no/natur-og-miljo/artikler-og-publikasjoner/the-norwegian-emission-inventory-2013 (2013).

8. Standard Norge. NS-3059. Enclosed wood heaters. Smoke emissions. Requirements. Standard Norge (1994).

9. NVE. Norwegian Water Resources and Energy Directorate (NVE). Available from: www.nve.no.

10. Bauer C. Holzenergie [Bioenergy]: Paul Scherrer Institut Villigen, Swiss Centre for Life Cycle Inventories (www.ecoinvent.ch) (2007).

11. European Environment Agency (EEA). *EMEP/EEA air pollutant emission inventory guidebook 2013. Technical guidance to prepare national emission inventories* (2013)..

12. Ministry of Climate and Environment (KLD). *Forskrift om begrensning av forurensning (forurensningsforskriften) [Pollution control regulation]* (2010).

13. Bond, T. C. *et al.* A technology-based global inventory of black and organic carbon emissions from combustion. *Journal of Geophysical Research: Atmospheres* **109** (2004).

14. Tomter S. M., Hylen G., Nilsen J.-E. Ø. Development of Norway's national forest inventory. In: Tomppo E, Geschwantner T, Lawrence M, McRoberts RE (eds). *National forest inventories. Pathways for common reporting*. Springer, Heidelberg, pp 411-424 (2010).

15. Nurminen T., Korpunen H., Uusitalo J. *Time consumption analysis of the mechanized cut-to-length harvesting system*, http://www.silvafennica.fi/article/346 (2006).

16. Vennesland B, Hohle AE, Kjøstelsen L*. Prosjektrapport klimatre. Energiforbruk og kostnader - Skog og bioenergi [Project report climate tree. Energy use and costs - Forest and bioenergy]*; Norwegian Institute of Bioeconomy Research. http://www.skogoglandskap.no/filearchive/rapport_14_13_prosjektrapport_klimatre_energiforbruk_og_kostnader_skog_og_bioenergi.pdf (2013).

17. Belbo, H., Talbot, B. & Kjøstelsen, L. Systemanalyse av ti forsyningskjeder for skogflis basert på heltrevirke [System analysis of ten supply chains for wood chippings based on roundwood], Norwegian Institute of Bioeconomy Research, http://www.skogoglandskap.no/filearchive/rapport_21_12_systemanalyse_av_ti_forsyningskjeder_for_skogflis_basert_pa_heltrevirke.pdf (2012).

18. Ranta, T. & Rinne, S. The profitability of transporting uncomminuted raw materials in Finland. *Biomass and Bioenergy* **30**, 231-237 (2006).

19. Spinelli, R. & Hartsough, B. A survey of Italian chipping operations. *Biomass and Bioenergy* **21**, 433-444 (2001).

20. Spinelli, R., Magagnotti, N., Paletto, G. & Preti, C. Determining the impact of some wood characteristics on the the performance of a mobile chipper. *Silva Fennica* **45**, 85-95 (2011).

21. Brunberg, T. Fuel consumption in forest machines 2012. Skogforsk report no. 789-2013; Forestry Research Institute of Sweden. http://www.skogforsk.se/contentassets/b89024b19fbf42a599fbfc0c4c322341/bransleforbrukningen-hos-skogsmaskiner-2012---arbetsrapport-789-2013.pdf (2013).

22. Ecoinvent. Life cycle inventory database v2.2. Swiss Centre for Life Cycle Inventories (2010).
